# Wills’ Hospital

**Published:** 1843-03-04

**Authors:** George H. Burwell

**Affiliations:** Resident Physician


### CLINICAL LECTURES AND REPORTS.

### WILLS' HOSPITAL.

**CLINIC OF DR. ISAAC PARRISH.**

[Reported by George H. Burwell, Resident Physician.]
*Jan.* 25, 1843.

CASE I.—*Scrofulous Conjunctivitis—ulcers of the cornea—photophobia, &c.*—Jan. 25, James McIntosh, aged 16 months, was presented by his parents for advice, on account of a severe attack of scrofulous conjunctivitis. They stated, that about Christmas, the child "took cold" which fell into the left eye: at the end of a week the right eye was attacked. The eyes have been much inflamed since that time, with intolerance of light, and lachrymation. There is a vesico-pustular eruption about the eyelids and cheek. The child is fretful and feverish, sleeps but little, and is cutting several large molar teeth. The bowels are constipated, and the discharges small, slimy, and greenish.

The general aspect of the infant is strumous. No regular course of treatment has been pursued in this case—the parents have relied mainly upon the advice of their friends in using washes, drops, &c.

℞. Calomel, grs. iv.

Pulv. Ipecac, gr. j.

Mft. pulv. No. ij. One to be taken to-night, and the other on the night of the 27th, to be followed each morning by a small table spoonful of Syrup Rhei.

℞. Sol. Nitrat. Argent, grs. ij.
to ℥j. of water.

Apply a few drops to each eye every morning.

January 28. Much improved, will permit the eyes to be examined without resistance; photophobia slight ; appetite better ; sleeps better than for some weeks past; the medicine has operated freely ; the discharges were first green and slimy, to-day quite yellow and natural.

℞. Sulph. Quininæ, grs. viij.

Elix. Vitriol, gtt. viij.

Syrup Sarsap. comp. f.℥ij.

Take a teaspoonful three time daily; give a tepid salt bath, twice a week, and continue drops to the eyes.

February 1st. Is still improving; general health better; scarcely any intolerance of light. Continue treatment, and keep the bowels open with syrup of rhubarb; weak animal broth once a day, in addition to milk diet.

This case offers a fine example of the disease known as scrofulous conjunctivitis, and may be taken as a sample of a class very numerous in practice. A class which constitutes a large proportion of the cases of ophthalmia occurring in childhood. Mackenzie rates the number as high as 90 out of 100—that is out of 100 children who may be affected with inflammation of the eyes, 90 will present the strumous character. I do not know whether the statistics of this house would justify so high a ratio, but speaking from a general impression, I should think they would. The disease is marked by several striking features, to which I would call your attention.

In the first place we perceive the external marks of the scrofulous diathesis, strikingly depicted The infant has a pallid and bloated countenance, light hair, tumid upper lip, and large abdomen, with disordered bowels; the skin is flabby and inactive. the extremities are apt to be cold, and the chain of absorbent glands along the sides of the neck are hard and enlarged.

In regard to the eyes we have first to remark the peculiar expression of the countenance caused by the spasmodic closure of the orbicularis muscle, and the consequent contraction of the other muscles of the face. The head is bent forward upon the breast, and every attempt to raise it, is firmly resisted by the child. This peculiar physiognomy and the bent position of the head, are alone sufficient to characterize the disease, and enable us to decide upon its character the moment we see the patient.

They indicate the existence of what is technically termed photophobia scrofulosa; an intolerance of light arising from an irritation of the retina, which is probably sympathetic of derangement of the digestive organs, or which may arise from that general nervous irritability which attends the anæmic condition of the system, in which we find this class of subjects. This photophobia is attended with epiphora, and not unfrequently with fits of sneezing, and copious mucous discharges from the schniderian membrane, showing the sympathy which exists between these several parts, dependant in a great measure upon the distribution of the lachrymal nerve; which, after supplying the lachrymal gland is distributed to the orbicularis palpebrarum, and to the conjunctiva.

Photophobia to a greater or less extent is so constant in scrofulous ophthalmia, as to be considered an element of the disease. It does not appear to be owing to an inflammatory condition of the retina, or to a structural lesion in any part of the eye.

As an evidence of the soundness of the retina, we find that the perception of objects is as acute as in health, provided the light be so moderate as not to dazzle the eye; thus we often see children, who, during the whole day will lie with their heads buried in a pillow, or will seek a dark corner in a room, and scream if the least ray of light is admitted to them, who will brighten up in the evening, and play with their toys by candle light, with as much delight and with as keen vision as in health. It is also true that in those cases where the photophobia is most severe the external tissues of the organ are often but slightly injected, thus affording additional confirmation to the view.

It must be borne in mind, however, that generally speaking, the cornea presents specks or pimples, in those cases which exhibit the photophobia, and that when the pustules are large the photophobia is generally less severe than in those cases where they are small.

Upon the right cornea of this infant, near its centre, you observe a small ulcer, into which a fasciculus of vessels enters; this is the result of a pustule which has discharged itself. It is not uncommon to see five or six pustules, or ulcers, upon one eye, giving to the conjunctiva the appearance of being covered with an eruption. You are aware that this membrane is a sort of intermediate tissue between skin and true mucous membrane; and that it may be affected with diseases common to both of these structures. Hence the several varieties of scrofulous ophthalmia have been described under the terms of exanthematous, porriginous, pustular, phlyctenular and apthous ophthalmia; they are all, however, essentially of the same nature, and require a like treatment. In this case the co-existence of a vesicular eruption around the eyelids and upon the cheek, would indicate the eruptive character of the complaint.

Bear in mind, then, that this is an eruptive ophthalmia, that it affects the conjunctiva as a part of the skin, and not as a mucous surface, and that the pimples which characterize it, vary in size from an opaque spot, which is barely discernible to the size of a pin's head.

Beer, the German ophthalmologist, has laid much stress upon the distinction between the small and large pustules. The former are called phlyctenulæ, and the latter pustules. The phlyctenular variety is perhaps the most severe and obstinate, but I apprehend that the distinction is not of much practical importance.

The mode of termination of the eruption of scrofulous ophthalmia, is a matter of much importance to the vision of the patient. The most favourable method is by absorption, which often happens, if the case is mild, and if remedies are applied early. In many cases, as in that before us, the pustule bursts, and a little pit or ulcer appears, which under favourable circumstances heals rapidly, leaving behind a white speck, which, if the inflammation be subdued, and if the previous ulcer has been superficial, soon disappears. In less favourable cases the ulcer penetrates or burrows into the laminæ of the cornea, inducing excessive irritation for a long period, and at last heals with a permanent pearl colored cicatrix, which if it occur in front of the pupil, offers a serious and irremediable obstruction to the passage of light towards the retina. In a few instances the cornea is completely penetrated, the aqueous humour escapes, and the iris falls forward into the opening, to the edges of which it contracts adhesions—forming synechia anterior.

In the large proportion of cases, however, scrofulous ophthalmia runs its course without the occurrence of permanent opacity, or other serious structural lesion. The disease may persist with greater or less severity for months or even for years, one relapse may follow another, with the most obstinate regularity, until the friends of the little patient and the physician, have lost all patience and almost abandoned hope, and yet the eyes will come out of each attack, without serious injury. This fact has been repeatedly verified in the practice of this hospital. Patients discharged cured, after a long and tedious attack, are returned upon us in a few weeks as bad as ever, and go through the same ordeal, to be again discharged and again returned. And yet we see them ultimately cured with good vision.

I have had under my care, for three years past, an intelligent and sprightly lad, who, during most of the time. has been the subject of scrofulous ophthalmia; he has had many successive crops of pustules and ulcers, and has repeatedly suffered for weeks together from the most distressing photophobia; and yet his eyes have escaped permanent opacity, and the sight is, at times, as perfect as natural.

There is another observation upon this point which it may be well to bear in mind, and that is, that in those cases where the photophobia is the most intense, the danger of permanent mischief by perforation of the cornea, &c., is, as a general rule, slight. This is probably owing to the fact previously stated, viz.; that the greatest intolerance of light occurs in cases where the corneal specks are small and numerous, and not single and deep seated.

The treatment in the case before us, has been both constitutional and local. The state of the secretions first claimed our attention. With a view of correcting these we directed the calomel and ipecac.

This was followed by a rapid improvement in the condition of the eyes; healthy discharges being restored, the way was prepared for tonics—the most efficient of which, in this disease, is sulph. quiniæ. Added to this the salt bath and frictions, with a view of establishing an active capillary circulation, and an improvement in the nutriment of the child, constitute the main features of the treatment. The effect, so far as the case has progressed, is very striking—the child having improved more under this treatment in a few days, then it did in as many weeks before. We shall hope, in a short time, to see the cure established.

There are many important practical considerations connected with the treatment of this complaint, which cannot now be entered into. The principle which should constantly be borne in mind is, that the disease has a constitutional origin, and that it is to be reached through the general system. Constant teazing of the eyes, by frequent efforts to examine them, or by making repeated applications to them, is very injurious. Confinement in a dark room, repeated leeching, and low diet, cannot, we think, be too strongly reprobated. On the other hand, exposure to the fresh air, nutritious diet, salt bathing—with alteratives and tonics, administered according to the indications present, constitute the general outline of the plan of treatment most approved and successful.

